# Offset curves: An application in road simulation

**DOI:** 10.1371/journal.pone.0309698

**Published:** 2024-11-05

**Authors:** Aqsa Rasheed, Uzma Bashir

**Affiliations:** Department of Mathematics, Lahore College for Women University, Lahore, Pakistan; Air University, PAKISTAN

## Abstract

Routable Digital Maps represent a recent advancement driven by the growth of Intelligent Transportation System applications. Efforts have been made to develop and utilize maps within computer systems that can autonomously identify optimal routes. Typically, online map visualizations lack essential information such as traffic patterns, construction zones, or lane counts, which are crucial for designing accurate and realistic routes. This research introduces a route planning method that aggregates and reuses various data points to establish a robust framework for developing new routing plans. The primary goal is to analyze the collected data and create a framework for estimating a geometrically smooth centerline of a road map route. This method involves generating a series of parallel curves, known as offset curves, to facilitate the creation of new routes and alternative lanes. The following examples illustrate some empirical results obtained from this approach.

## 1 Introduction

Roadways are an essential part of human civilization and various types of roads combine to construct road networks that serve as the backbone of contemporary society [1]. With advancements in computing technology, road models have become extensively employed in a variety of applications such as games environments and virtual worlds [2]. With the rising needs, planning and developing road maps requires special attention. Road networks are significant not just in various virtual worlds, but also in urban modeling, planning, intelligent transportation systems, vehicle path planning and driving simulation [3].

The advancement of intelligent vehicle technologies has raised the demand for a high-precision road map. Navigation or geographic information systems alone are insufficient to meet the growing demands of intelligent vehicle systems such as self-driving cars [4]. A method for developing realistic and effective lane-level road maps for intelligent and autonomous vehicles [5] proposed a novel method for dynamically producing high precision road maps using statistical analysis of GPS traces in vehicles.

High-fidelity 3D road network models are required by many computer applications, including racing games and driving simulators. Furthermore, only a few approaches exist for automatically generating 3D plausible road networks, particularly those in the actual world. A unique method presented [6], for converting current road geographical information system (GIS) data with just 2D road centerline information into high-fidelity 3D transportation infrastructure models.

Navigation systems aid practically any physical movement, including sailing, flying, hiking, cycling, and driving. Traces provided by global positioning systems (GPS), may monitor the exact time and accurate coordinates of moving objects. Route planning based on GPS data and current location produces a single source shortest path [7], centered on Dijkstra’s algorithm. Likewise, an intranet for aided cognition that can develop personalized maps for each individual and extrapolate their everyday activities and travels from organic GPS data was described [8]. A spatial model for estimating vehicle miles traveled was proposed based on large GPS data streams [9], which is an essential metric utilized by both federal and regional highway authorities in the United States for transit planning. An approach exclusively described for generating a two-lane path in 2.5D configurations [10], by translating a collision-free 2D two-lane road in multidimensional space onto a terrain graph surface.

Urban road networks are crucial for city transportation, facilitating both human and goods movement, and supporting various Location-Based Services (LBS) such as vehicle route planning and navigation. These functions are vital for smart mobility. Despite their importance, creating and updating road networks efficiently and cost-effectively remains a significant challenge. For example [11], introduced a data-driven method for extracting and updating road elements, such as road centerlines, using large-scale GPS data.

The methodologies described are based on visualizing data from diverse sources, represented through curves and surfaces. This paper focuses on the use of curves in road map modeling. Previous efforts in road modeling include work on [12, 13], and path design using variable degree B-splines [14]. While B-splines are versatile, they have limitations, including generating wiggles at the data endpoints, failing to preserve the geometrical features of data, and incurring high processing costs.

The work given here is based on Bezier-like curves, which have pleasant parameter optimization qualities. A generalization of Bezier-like curves based on trigonometric polynomial basis functions is developed and used to simulate road maps and path planning. The contribution of the paper are summarized as follows:

1. Construction of road curves
2. Estimation of centerline
3. Lane and outliers determination

## 2 Background and definitions

Road curves are extremely important in the geometric design of road and railway alignments. As a result, it must be thoroughly researched and constructed in order to ensure safety, comfort, and convenience when operating automobiles or trains on road bends. Curves are the geometrical arcs formed by changes in road alignment or gradient.

A straight road or track is usually preferable since it saves money on construction, transit, and maintenance. However, when the alignment or grade of a road or railway track changes, it becomes necessary to create bends under the different circumstances such as excessive cutting or filling, natural or artificial obstruction and to create diversions.

Road curves serve a variety of purposes in the alignment of a road or railway track. Curves can be used to provide a gradual shift in direction or orientation in the alignment. Curves are built to facilitate turning on roads and tracks.

### 2.1 Types of road curves

There are two types of road curves namely horizontal and vertical road curves.

#### 2.1.1 Horizontal curves

Horizontal curves are road curves that are supplied at turning locations to allow for a progressive shift in the direction of alignment of a road or rail. These curves are often in the horizontal plane. For a wide gauge railway track, the minimum radius of a horizontal curve should be 175 m. Curves on a road, both horizontal and vertical, must assure traffic safety, comfort, and convenience.

1. Simple road curves: A simple curve is one that consists of a single arc of a circle to which two straight tangents join and cause a deviation of the road through an angle *theta*.
2. Compound road curves: A compound curve is a series of two or more simple curves of various radii bending in the same direction.
3. Reverse curves: The reverse curve is formed by two simple curves with equal or differing radii turning in opposite directions. The two curve centers are on opposing sides of a common tangent.
4. Transition curves: The transition curve is defined as the curve whose radius progressively goes from infinity to a finite value equal to that of the circular curve to be linked and vice versa.

#### 2.1.2 Vertical curves

Vertical curves are the curves that are supplied in the alignment of a road or rail with a change in grade. There are two types of vertical curves listed below.

1. Crest vertical curves (Summit curves): These are the curves that are supplied in the alignment of a road or rail with a change in gradient.
2. Sag vertical curves (Valley curves): Valley curves are road bends that have a convex surface on the descending side.

## 3 Methodology

### 3.1 Mathematical representation for generalized trigonometric functions

A set of trigonometric basis functions of degree *n* ≥ 4 is proposed to define Bézier curves of various degrees. The trigonometric blending functions of degree *n* ≥ 4, with two shape parameters, for $\varphi \in [0,\frac{\pi }{2}]$ are defined as [15]:

$$\begin{array}{c} B_{j,n}\left(\varphi \right)=\{\begin{array}{cc} \text{sin}\left(\varphi \right)B_{j-1,n-1}\left(\varphi \right)+\left(1-\text{sin}\left(\varphi \right)\right)B_{j,n-1}\left(\varphi \right) & j=0,1,2,\ldots ,\lfloor n/2\rfloor -1\\ \text{sin}\left(\varphi \right)B_{j-1,n-1}\left(\varphi \right)+\text{cos}\left(\varphi \right)B_{j,n-1}\left(\varphi \right) & j=\lfloor n/2\rfloor \\ \left(1-\text{cos}\left(\varphi \right)\right)B_{j-1,n-1}\left(\varphi \right)+\text{cos}\left(\varphi \right)B_{j,n-1}\left(\varphi \right) & j=\lfloor n/2\rfloor +1,\ldots ,n-1,n \end{array} \end{array}$$ (1)

where $B_{j,n}\left(\varphi \right)=0$ for *j* = −1 and *j* ≥ *n*. And the recursive relation is generated by using 3rd degree polynomial functions with two shape parameters [16]. The trigonometric basis functions $B_{j,n}\left(\varphi \right)$ defined in Eq 1 hold the following geometric properties:

1. sum to unity: $\sum_{j=0}^{n}B_{j,n}\left(\varphi \right)=1.j=0,1,2,\ldots ,n$
2. non negativity: $B_{j,n}\left(\varphi \right)\geq 0$,
3. symmetry: These basis functions are symmetric with respect to the parameters *φ* and $\left(\frac{\pi }{2}-\varphi \right)$.
  $$B_{j,n}\left(\varphi ,l,m\right)=B_{n-j,n}\left(\frac{\pi }{2}-\varphi ,m,l\right),j=0,1,2,\ldots ,n$$
4. monotonicity: For the specified values of shape parameters *l* and *m*, the basis function $B_{0,n}\left(\varphi \right)$ decreases and $B_{n,n}\left(\varphi \right)$ increases monotonically.

**Fig 1:**
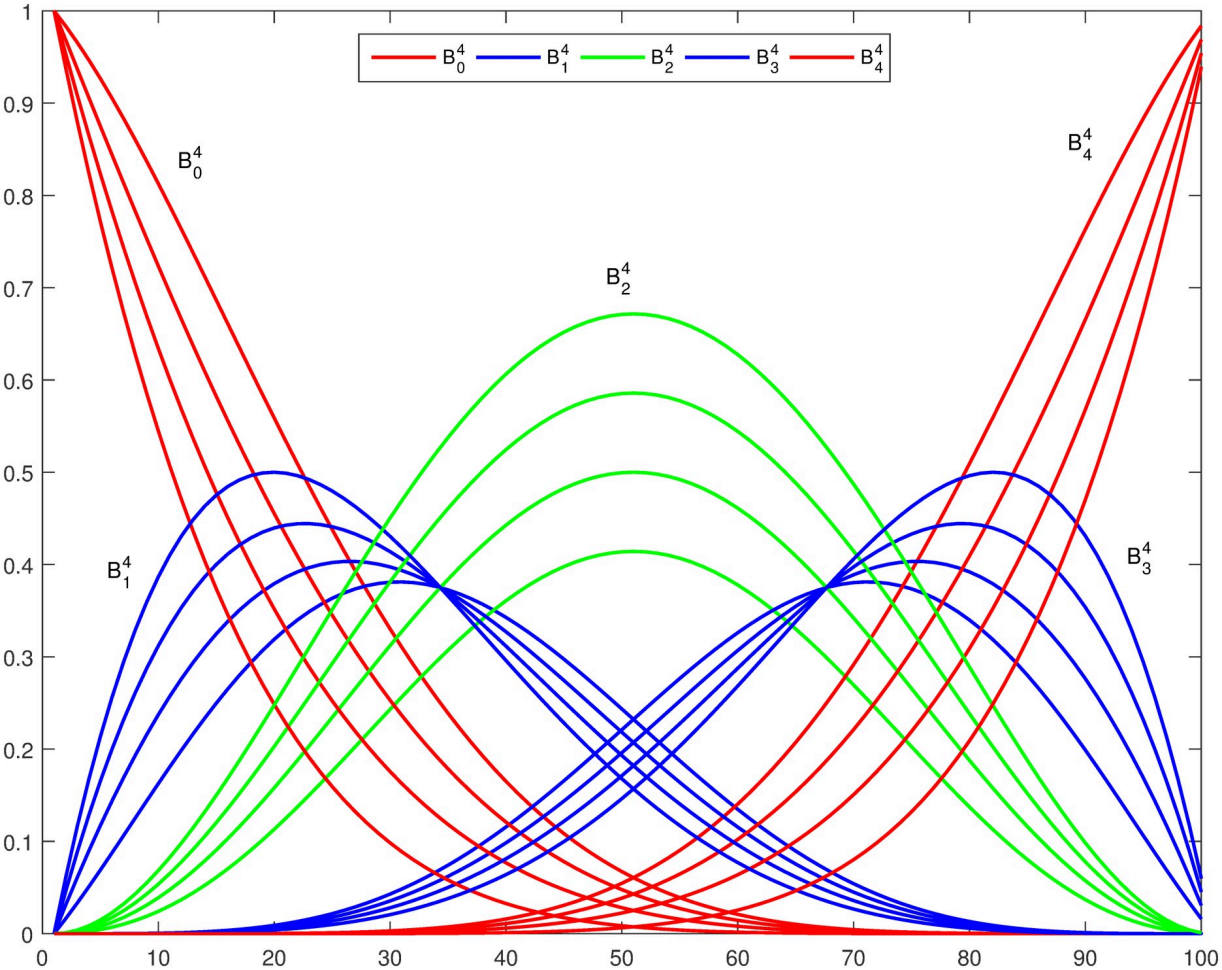
Trigonometric basis functions with *n* = 4, *l* = *m* = −1, 0, 1, 2.

**Fig 2:**
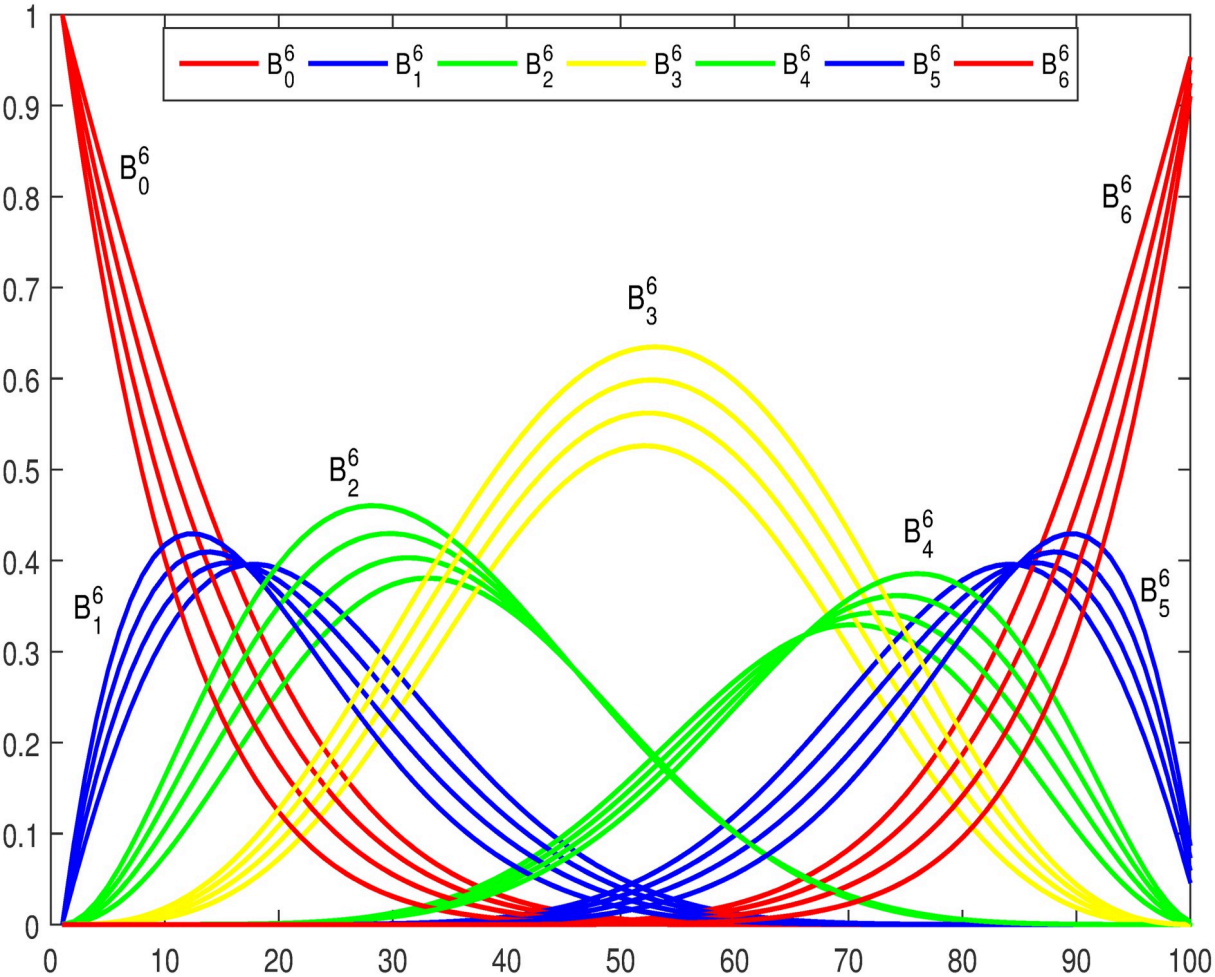
Trigonometric basis functions with *n* = 6, *l* = *m* = −1, 0, 1, 2.

Figs 1 and 2 show a graphical representation of trigonometric basis functions for *n* = 4 and 6 with different shape parameters. It can be envisioned that these basis functions satisfy all the properties listed above.

### 3.2 Shape analysis of trigonometric Bézier curves

Bézier curves have the following parametric representation based on the control points and basis functions.

$$\begin{array}{c} U\left(\varphi \right)=\sum_{j=0}^{n}B_{j,n}\left(\varphi \right)Q_{j},\;\varphi \in \left[0,\frac{\pi }{2}\right]. \end{array}$$ (2)

**Fig 3:**
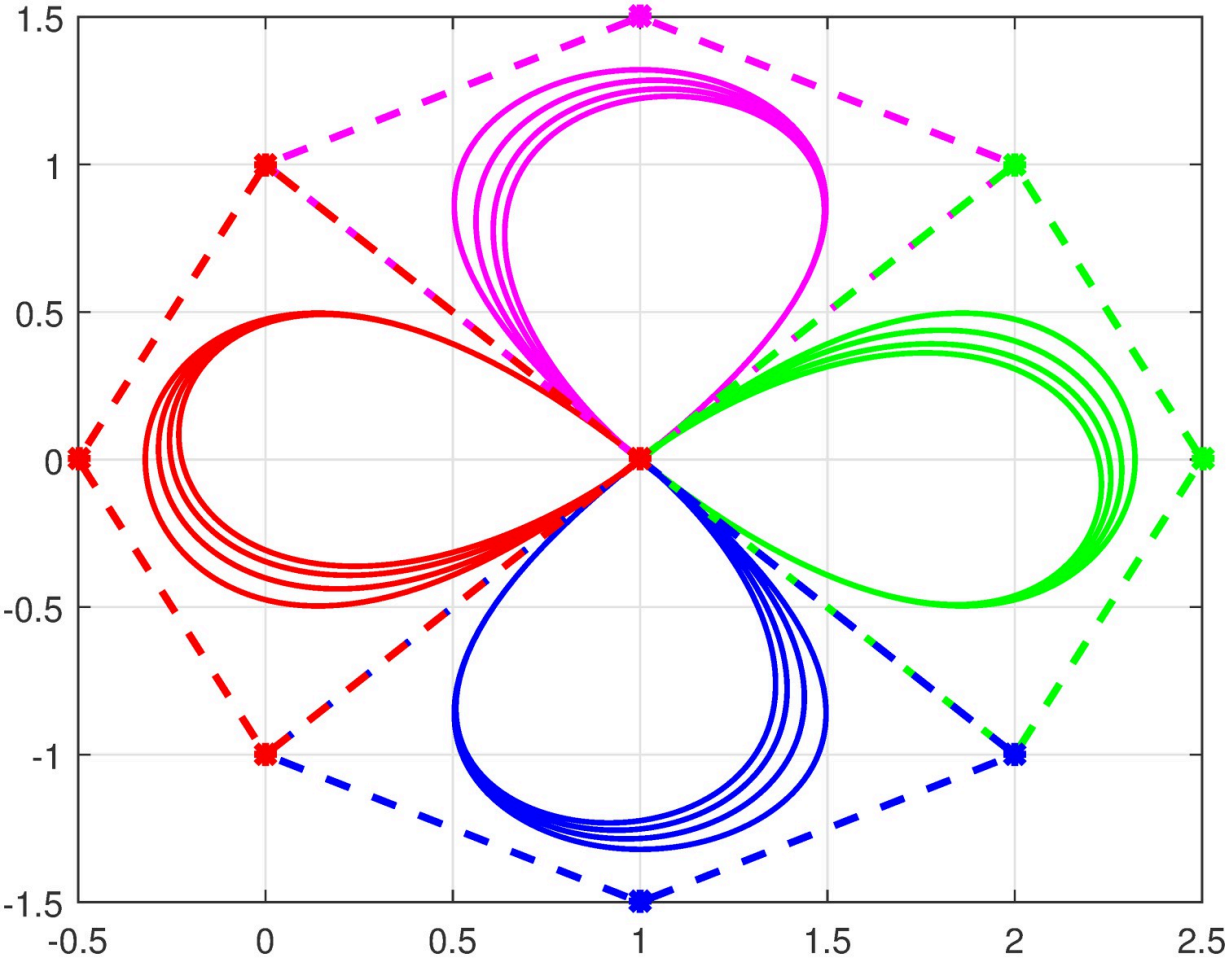
Trigonometric Bézier curve with *n* = 4, *l* = −1, 0, 1, 2, *m* = 2.

**Fig 4:**
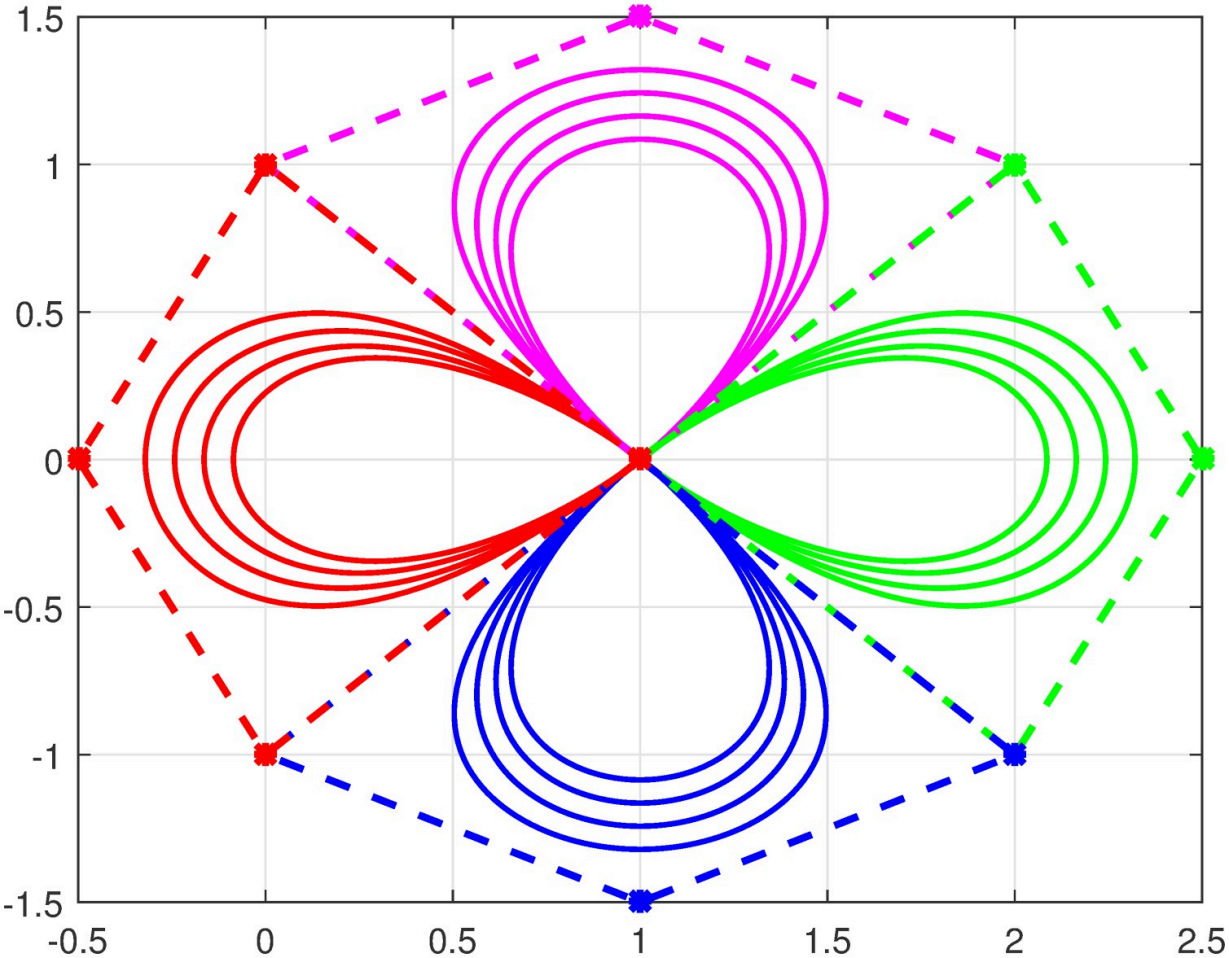
Trigonometric Bézier curve with *n* = 4, *l* = *m* = −1, 0, 1, 2.

Here U(φ) defines a Bézier curve of degree *n* ≥ 3 with trigonometric functions $B_{j,n}\left(\varphi \right)$ defined in Eq 1 and $Q_{j}=\left(x_{j},y_{j}\right)$ are control points. The defined curve in Eq 2 satisfies all the properties of parametric Bézier curves specifically, endpoint interpolation, convex hull, geometric invariance, symmetry and shape adjustable property. Figs 3 and 4 represent four closed quartic curves with different combinations of shape parameters.

## 4 Offset modelling

The offset O(φ) of a parametric curve U(φ)=(x(φ),y(φ)), is defined as the set of all points that lies at a constant distance *d* known as offset distance, in the direction of normal to the curve [17], written as

$$\begin{array}{c} O(\varphi )=U(\varphi )\pm dN(\varphi ) \end{array}$$ (3)

where N(φ) represents the normal vector of the curve

$$\begin{array}{c} N(\varphi )=\frac{\left(-y^{\prime }\left(\varphi \right),x^{\prime }\left(\varphi \right)\right)}{\sqrt{x^{\prime }{\left(\varphi \right)}^{2}+y^{\prime }{\left(\varphi \right)}^{2}}}. \end{array}$$ (4)

The offset curve defined in Eq 3, is not a rational curve. The square root function in the normal vector is a major drawback of these curves in the applications of computer-aided design and manufacturing (CAD/CAM). To overcome this problem [18], a class of PH curves which have a rational description for their normal is introduced. But working with PH curves reduces the degree of freedom. An interpolation technique [19], based on the trigonometric Bézier curve is considered for the interpolation of curves as well as their offsets.

**Fig 5:**
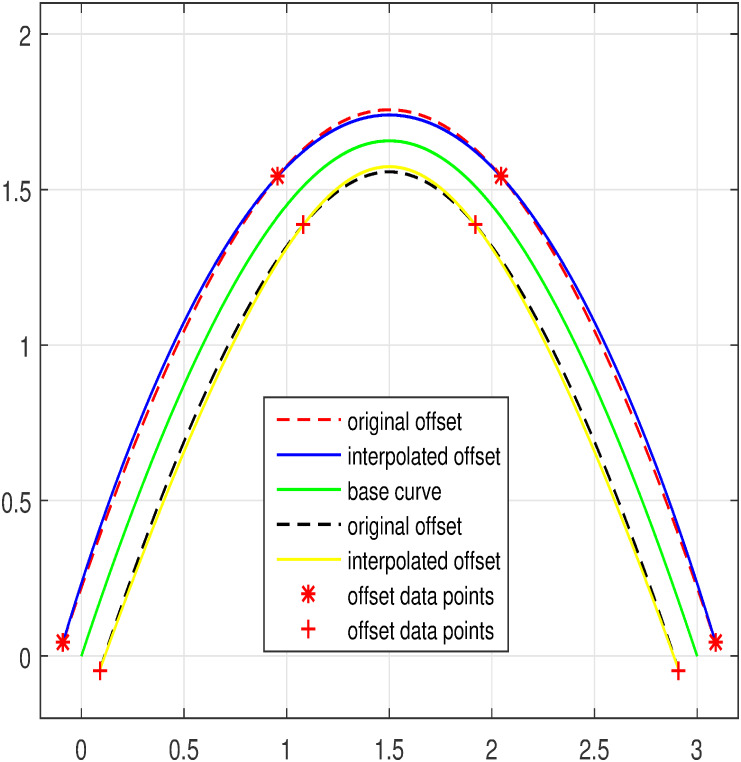
Degree three offset interpolation.

**Fig 6:**
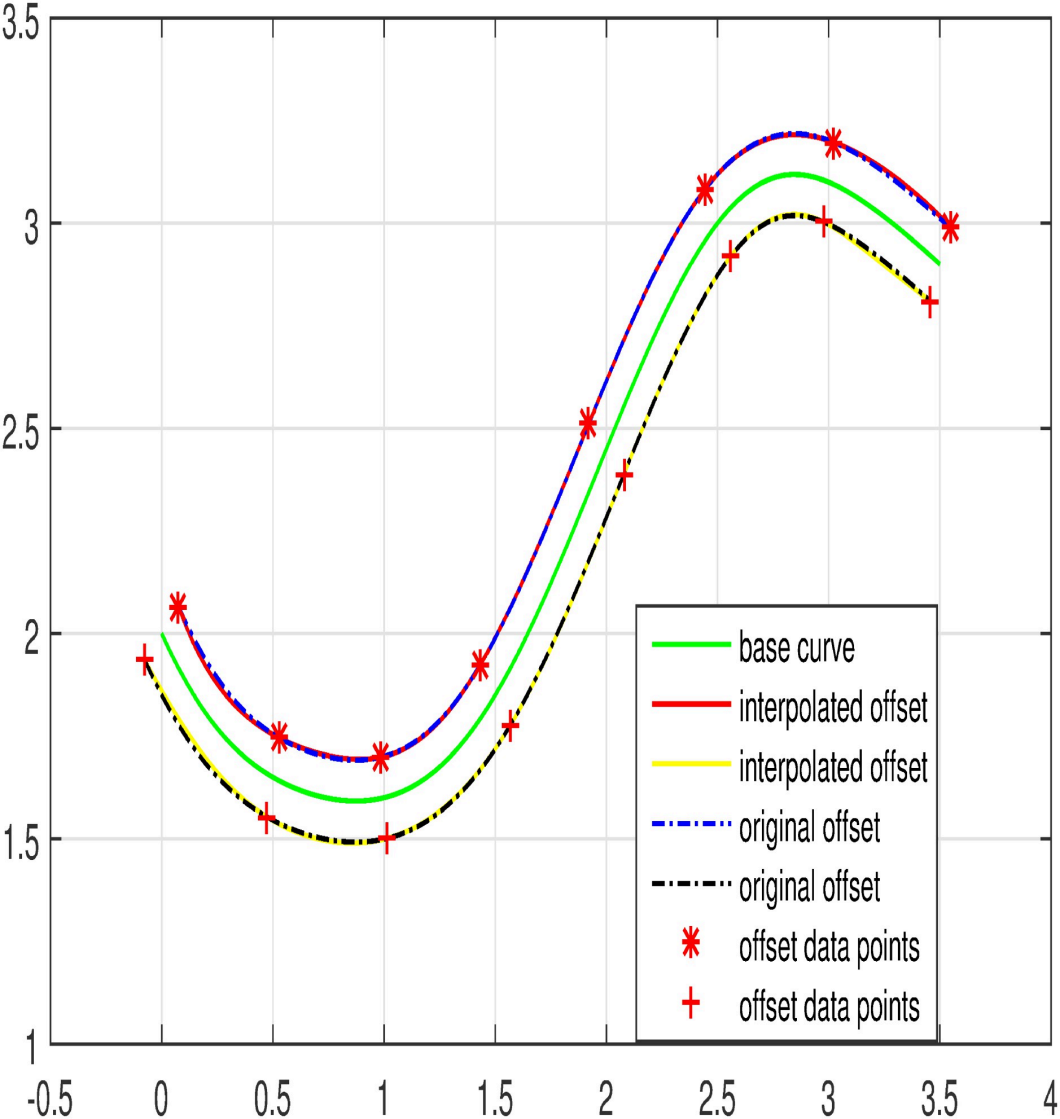
Degree seven offset interpolation.

**Fig 7:**
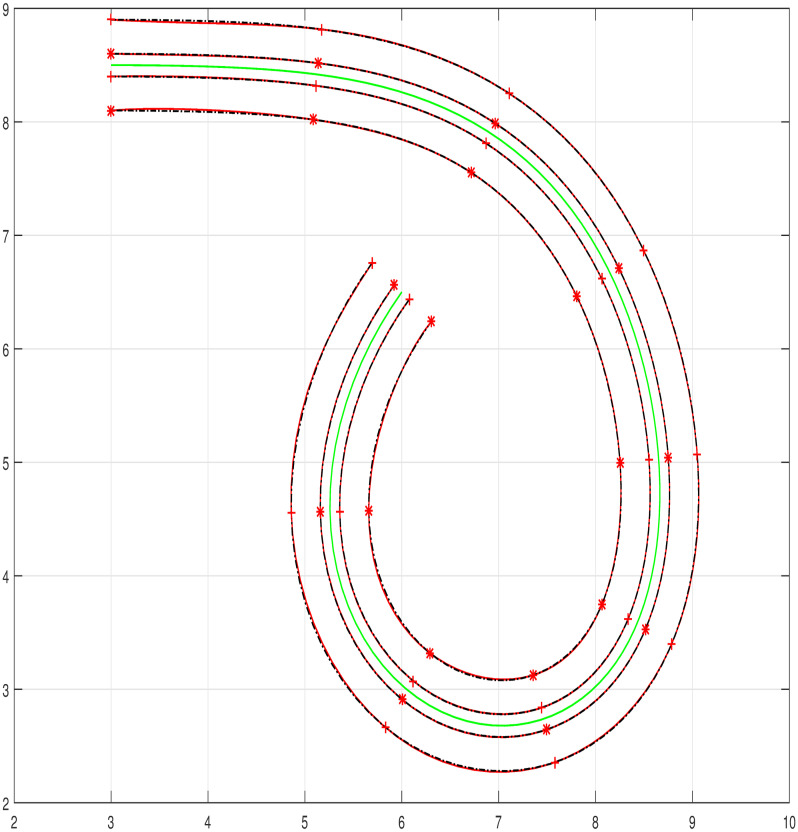
Degree nine offset interpolation.

The input data is taken from the offset curve and interpolated by means of trigonometric Bézier curve of any degree depending on the set of data points. Few examples are considered where curves and their offsets are used for modeling. Figs 5–7 represent the curves and their corresponding inner and outer offset curves. The interpolation of curves and their offsets are promising for modeling as well as for practical application Table 1.

**Table 1:** Data for examples.

Degree of curve	Offset distance	Degree of curve	Degree of interpolated offset curve	Max Error
	*d*	U(φ)	O(φ)	$\epsilon =max|O\left(\varphi_{i}\right)-U\left(\varphi_{i}\right)|$
Three	±0.1	3	3	0.0034
Seven	±0.2	7	7	0.01
Nine	±0.1, ±0.4	9	9	0.0022, 0.0088

## 5 Application in road simulation

This section is concerned with the analytic representation of road segments, which are converted into 2D road geometric models composed of road curves, more specifically two-dimensional centerline and outlier models. The geometric modeling of road segments is based on numerous road properties, such as road centerline locations, normal circular slope, super-elevation, traffic volumes, and side trajectories. The analytic road network interpretation generates discrete points along the road centerline. Using these points as vertices, a parametric expression for the road curve is constructed. Furthermore, multiple parallel curve representation gives an estimation for lane count and outliers.

### 5.1 Road segments

The suggested method is intended for modeling and simulation specialists, such as video game developers, and other digital systems that require realistic roadway simulations. The objective is to provide quick and efficient 2D road modeling for high-fidelity road framework in applications such as racing games and driving simulations.

The study and modeling of interchanges is a critical component of simulations. Road map interchange provides a wide range of road segments and is an important aspect of modeling. The flow of traffic is intimately related to the city interchanges, which serve as a traffic junction for inlets and outlets. A case study of an interchange of the city Lahore in Pakistan, shown to highlight the necessary aspects such as centerlines and outliers. The heads up digitization [20] method is used to extract data from any map image.

#### 5.1.1 Simple road curve

**Fig 8:**
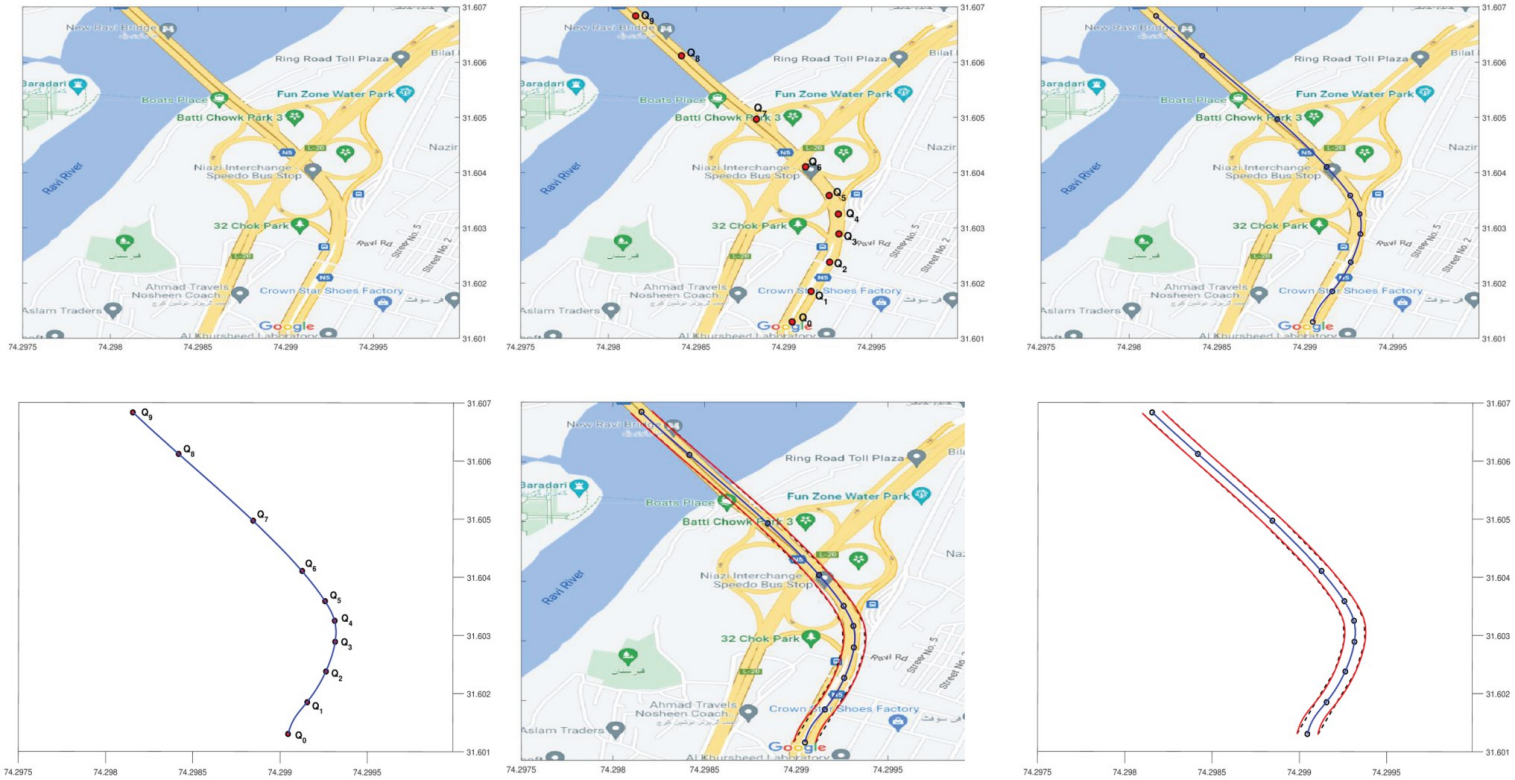
Simple road curve construction. (a) Google map image, (b) Extracted data points, (c) Centerline construction, (d) Centerline, (e) Interpolated offset, (f) Centerline and outliers.

In the first example, a simple road curve segment is selected on the map. A single nine degree curve is used to estimate centerline in Fig 8(a)–8(d), along with outliers represented in Fig 8(e) and 8(f).

#### 5.1.2 Reverse road curve

**Fig 9:**
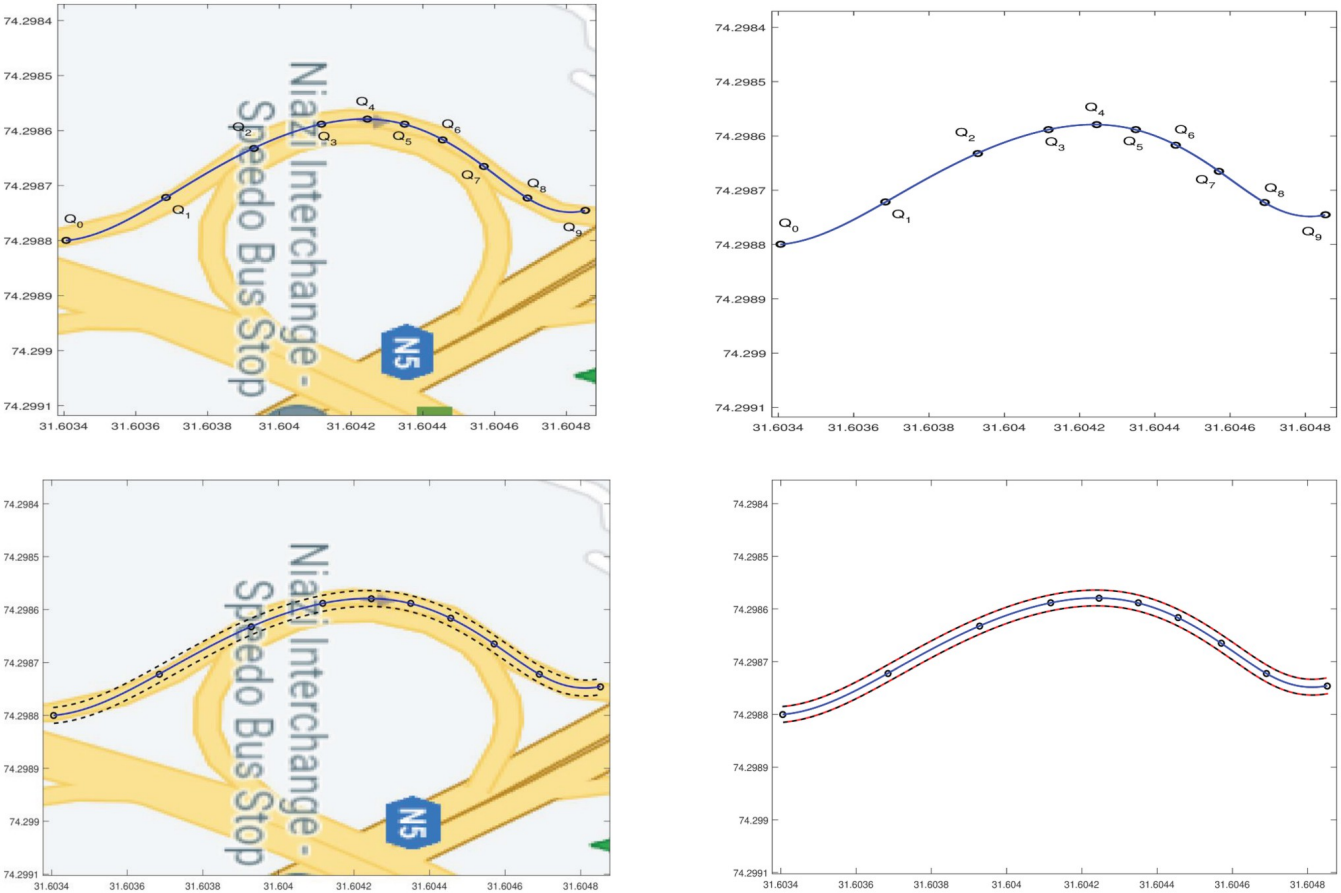
Reverse road curve construction. (a) Google map image, (b) Centreline, (c) Offset interpolation, (d) Centerline and outliers.

The next example is of reverse road curve where a single nine degree curve is used to estimate centerline along with outliers. The process is depicted in Fig 9.

#### 5.1.3 Compound road curve

**Fig 10:**
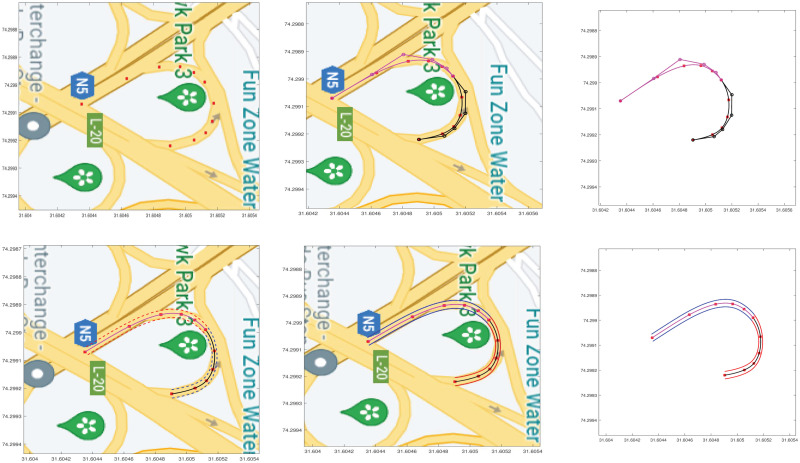
Compound road curve construction. (a) Selection of data points, (b) Joining of two segments, (c) Centerline construction, (d) Reference Offset curve, (e) Centerline and outliers, (f) Centerline and outliers.

A segment of the map is selected where a single degree curve does not estimate the centerline and its outliers. To overcome this problem joining of two five degree curves is discussed and shown in Fig 10(a). The centerline of the complete segment is represented with two curves and their outliers are represented by their corresponding interpolated offsets can be visualized in Fig 10(b)–10(f).

**Algorithm 1**: Construction of Road curves

1 All four techniques are summed up in the form of an algorithm for computation of approximated offset curve:

 **Input**: Choose number *k* = *n* + 1 of points (where *n* ≥ 3 is the degree of the curve) and error *ϵ*_*o*_.

2 Construction of centerline as a reference curve of the road map;

3 Construction of reference Offset lines as outliers of the road map;

4 Interpolation of offset lines;

 **Output**: centreline along with outliers of the road curve

5 Find the maximal deviation *d* = *max*|*A*_*i*_ − *S*_*d*_(*t*_*i*_)|;

6 **if** *d* ⩽ *ϵ*_*o*_ **then**

7  STOP (the result is defined by (4))

8 **end**

9 **else**

10  split the given curve into two segments;

11  Goto 1;

12  Interpolate offset curve *S*_*d*_(*t*) for each segment.

13 **end**

## 6 Conclusions

In this paper, a new automated method is proposed to reconstruct 2D highway curves from google map image. The method consists of two major steps: detection of road markings, and curve reconstruction (i.e., recognizing the types of curves and estimating the values of curve parameters). The method has been validated by experiments on a virtual google image data set. The virtual experiments showed that the proposed method achieve relative accuracy. The final parametric curves obtained using the method is confined by the highway geometric design standard.

Nonetheless, the proposed method takes advantage of the existence of a vast road data offers a unique, autonomous approach to 2D road network modeling, greatly decreases human costs, and benefits a wide range of road network-based applications. Future study will boost the effectiveness of the suggested technique, such as more reliable and precise modeling of complicated interchanges with three or more levels and road surface tracking of complex urban crossings.
